# Disruption of Retinol (Vitamin A) Signaling by Phthalate Esters: SAR and Mechanism Studies

**DOI:** 10.1371/journal.pone.0161167

**Published:** 2016-08-17

**Authors:** Yanling Chen, David H. Reese

**Affiliations:** Division of Molecular Biology, Office of Applied Research and Safety Assessment, Center for Food Safety and Applied Nutrition, U.S. FDA, 8301 Muirkirk Rd., Laurel, MD, 20708, United States of America; Laboratoire de Biologie du Développement de Villefranche-sur-Mer, FRANCE

## Abstract

A spectrum of reproductive system anomalies (cryptorchidism, hypospadias, dysgenesis of Wolffian duct-derived tissues and prostate, and reduced sperm production) in male rats exposed in utero to phthalate esters (PEs) are thought to be caused by PE inhibition of fetal testosterone production. Recently, dibutyl and dipentyl phthalate (DBuP, DPnP) were shown to disrupt the retinol signaling pathway (RSP) in mouse pluripotent P19 embryonal carcinoma cells in vitro. The RSP regulates the synthesis and cellular levels of retinoic acid (RA), the active metabolite of retinol (vitamin A). In this new study, a total of 26 di- and mono-esters were screened to identify additional phthalate structures that disrupt the RSP and explore their mechanisms of action. The most potent PEs, those causing > 50% inhibition, contained aryl and cycloalkane groups or C4-C6 alkyl ester chains and were the same PEs reported to cause malformations in utero. They shared similar lipid solubility; log*P* values were between 4 and 6 and, except for PEs with butyl and phenyl groups, were stable for prolonged periods in culture. Mono- and cognate di-esters varied in ability to disrupt the RSP; e.g., DEHP was inactive but its monoester was active while DBuP was active yet its monoester was inactive. DBuP and dibenzyl phthalate both disrupted the synthesis of RA from retinol but not the ability of RA to activate gene transcription. Both PEs also disrupted the RSP in C3H10T1/2 multipotent mesenchymal stem cells. Based on this in vitro study showing that some PEs disrupt retinol signaling and previous in vivo studies that vitamin A/RA deficiency and PEs both cause strikingly similar anomalies in the male rat reproductive system, we propose that PE-mediated inhibition of testosterone and RA synthesis in utero are both causes of malformations in male rat offspring.

## Introduction

Phthalates, alkyl or aryl esters of phthalic acid (referred to here as PEs), are non-covalently incorporated into polyvinyl chloride plastics during manufacturing to increase softness and flexibility of the finished product. They are also used in a wide variety of consumer products including cosmetics, personal-care products, adhesives, detergents, food packaging, medical devices, as excipients in pharmaceuticals, and in dietary supplements [1–3]. Because of their widespread use and tendency to leach from products, PEs have become pervasive in the environment. Metabolites of some PEs have been found in more than 97% of some NHANES urine samples indicating widespread human exposure [4]. Food is a significant source of exposure [3, 5]. Other important sources include water, personal care products, and cosmetics.

Evidence for widespread human exposure to PEs has raised human health concerns because of the similarities between congenital anomalies observed in the human male reproductive system and malformations in the reproductive systems of male rats exposed to PEs. Increased prevalence of cryptorchidism and hypospadias, two common congenital malformations of the male reproductive system, and a decline in sperm quality have been reported in some human populations [6–8]. Exposure of pregnant rats to PEs during late gestation causes a spectrum of malformations in the reproductive system of male offspring including cryptorchidism and hypospadias, dysgenesis of Wolffian duct-derived tissues (vas deferens, epididymis, seminal vesicles) and prostate, as well as reduced sperm production[9]. It should be noted that the adverse effects of PEs on the male reproductive system are not limited to exposure during development, but are also seen in pre-pubertal, pubertal, and adult rats exposed to PEs[9]. The adverse effects of PEs on the development of androgen-dependent reproductive tissues are generally attributed to low fetal testosterone (T) levels caused by PE interference with T synthesis by Leydig cells of the fetal testis [9, 10].

**Fig 1:**
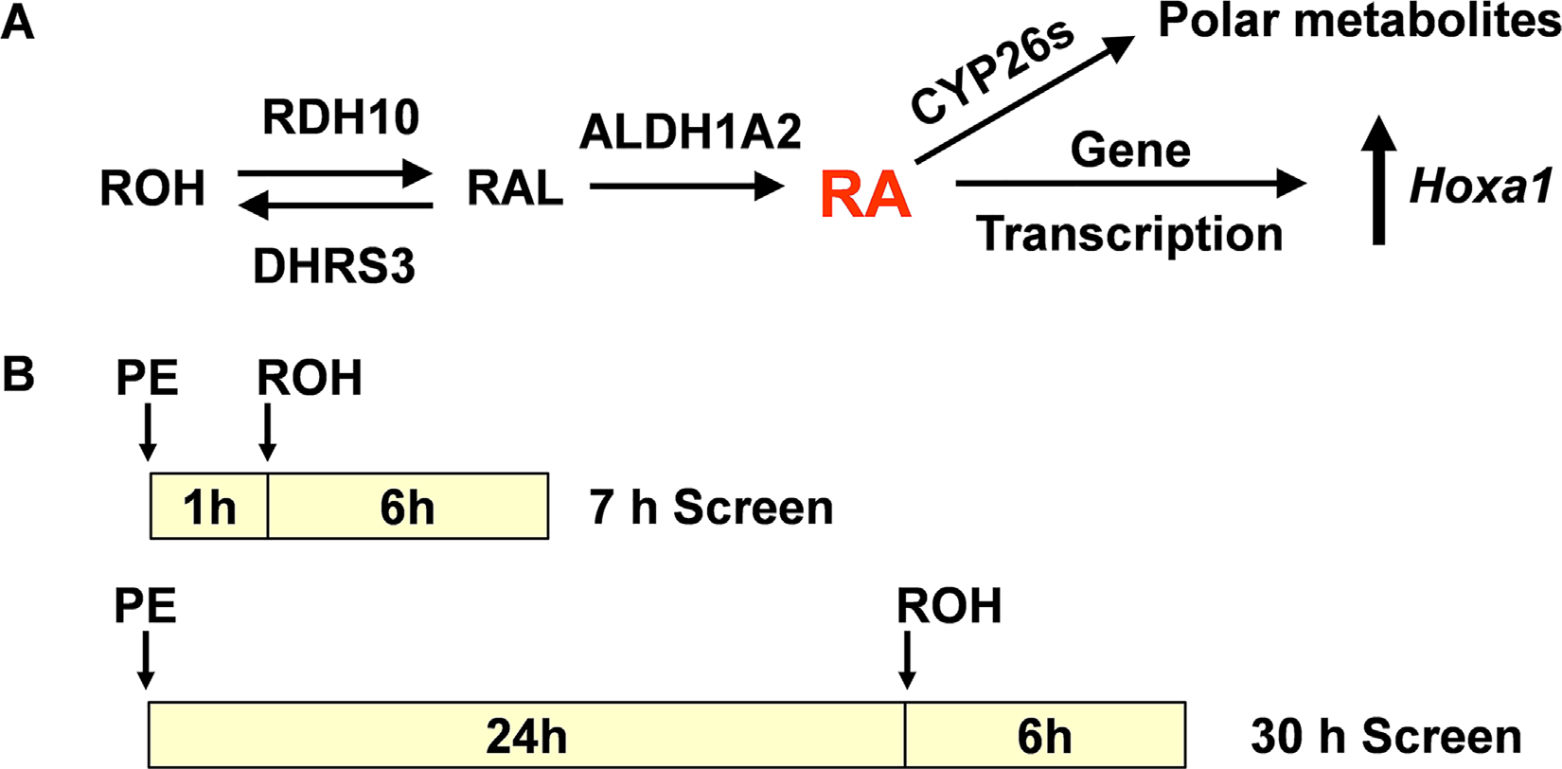
**(A) Major steps in the retinol signaling pathway of vertebrates. (B) Timeline for PE and ROH additions and duration of exposures for the 7 h and 30 h screens.** The initial PE concentration was 50 μM. After the addition of ROH, the PE and ROH concentrations were 45 μM and 0.3 μM respectively. The initial and final DMSO concentration was 0.5%. P19 cells were cultured in 96-well plates as described previously [11].

We recently described the development of a rapid cell based screen for identifying chemicals that disrupt the retinol (ROH; vitamin A) signaling pathway (RSP) [11]. This screen uses the mouse pluripotent P19 cell which can be induced to differentiate into cell types that are representative of all three embryonic germ layers. This cell line has a functional retinol signaling pathway [12] found in all vertebrates (Fig 1A) and, therefore, can metabolize retinol to its active metabolite, retinoic acid (RA), and maintain this compound at a concentration compatible with normal cellular function [13, 14]. RA is the activating ligand that controls the expression of a large number of protein-coding genes [15] and non-coding regulatory RNAs [16] by binding to, and activating, the RXR/RAR receptor complex on the retinoic response elements of RA-regulated genes. A well regulated retinol signaling pathway is essential for normal development of embryos and maintenance of cellular phenotype in adults in all vertebrates. Inadequate cellular levels of RA caused by vitamin A deficiency (VAD) or by interference with the synthesis of RA from vitamin A can cause abnormal development and loss of phenotype in adult tissues; abnormally high levels of RA caused by interference with its metabolism also can have adverse cellular effects [12].

The screen uses the level of ROH-induced expression of the homeobox gene, *Hoxa1*, as a measure of pathway function and the ability of a chemical to decrease or increase ROH-induced expression as an indication of pathway disruption. *Hoxa1* is rapidly upregulated by ROH, RAL, and RA in P19 cells [12]. It is one of numerous *Hox* genes regulated by retinol signaling in the mammalian embryo [17] where its expression is essential for normal hindbrain development and fetal survival [18]. In humans, homozygous mutations in *HOXA1* are associated with defects in brainstem, cardiovascular, and cognitive development [19, 20]. Chemicals that disrupt the retinol signaling pathway in this screen, therefore, have the potential to be teratogenic in rodents and humans.

In the original description of the screen [12], we reported that two phthalate diesters, DBuP and DPnP, significantly interfered with the retinol signaling pathway while a third, DEHP, had no apparent effect [11]. The monoester metabolite is thought to be the active form of PEs responsible for reproductive toxicity [21] yet these two diesters tested positive in the screen. Since PEs had not been shown previously to have an effect on retinol signaling, this unexpected mode of action prompted us to carry out a more extensive analysis of PE effects on the RSP to gain a better understanding of how members of this class of compounds affect the pathway. The goals of this study were: (1) to identify additional PE structures that interfere with the pathway to determine whether active compounds share structural and/or physicochemical properties that may provide insight into mechanisms of action in the P19 stem cells; (2) assess the relative stabilities of PE structures in culture; (3) compare the inhibitory effects of diesters with their cognate monoesters; (4) identify step(s) in the pathway that are inhibited by PEs; and (5) determine if PE inhibition of retinol signaling is unique to the P19 cell or if another cell type with a functional RSP is also affected.

## Materials and Methods

### Test Chemicals

For information on sources and purity of PEs used in this study, see S1 Table. Stock solutions were prepared in DMSO at a concentration of 50 mM and stored in the vapor phase of liquid nitrogen. Calculated values for log*P*, the physicochemical descriptor of lipophilicity (lipid solubility), were obtained from the ChemSpider Web site (http://www.chemspider.com). Some PEs were supplied as mixtures of isomers (S1 Table). The log*P* values used for these samples were based on the compound name designated by the supplier.

### Cell Culture and Assay Conditions

Mouse P19 pluripotent embryonal carcinoma cells [22] were obtained from the American Type Culture Collection (ATCC, Manassas, VA) and cultured in MEMα medium (Invitrogen, Carlsbad, CA) supplemented with 10% FBS (ATCC). The serum used in this study was from the same lot used previously [11] which had a retinol serum concentration of 73 nM; complete medium containing 10% serum, therefore, had a background retinol concentration not greater than 7.3 nM. All culture and assay conditions employed in the 96-well-format, P19-cell screen used for detecting chemicals that interfere with the retinol signaling pathway, including the MTT assay for cytotoxicity, direct cDNA synthesis from cell lysates, and quantitative real-time PCR using GAPDH as the reference gene for normalizing relative changes in gene expression by the 2 ^-ΔΔCt^ method, have been described in detail previously (11). The rationale for the use of a single PE test concentration of 45 μM was given previously [11] and is restated hear. Briefly, the screen was designed for prioritizing large numbers of chemicals for further studies (including dose-response analysis) so it was decided to use a single concentration initially to allow higher throughput. Citral was previously found to be a potent inhibitor (IC_50_ = 4.2 μM) of ALDH1A/RALDH2, the enzyme that converts RAL to RA in P19 cells (12) so all chemicals are initially screened at 45 μM, an approximately 10-fold higher concentration than the IC_50_ for citral, to allow detection of chemicals that are less potent than citral.

To ensure compound solubility in vitro, all PE stock solutions produced a final DMSO concentration of 0.4% in the medium. In the presence of added ROH, the final DMSO concentration was 0.5%. Untreated control cultures also contained 0.5% DMSO. The schedule of PE and ROH additions for the 7 h and 30 h screens are indicated in Fig 1B.

Mouse C3H10T1/2 mesenchymal stem cells, obtained from ATCC, were cultured in BME (Invitrogen) supplemented with 10% FBS (ATCC) as described previously [23] with the exception that the FBS was not heat-inactivated. Duplicate confluent monolayer cultures grown in 60-mm culture dishes containing 5 ml of medium were pre-treated for 1 h with DBuP or DBnP stock solutions or with DMSO only (control cultures). ROH was then added to PE-containing cultures and one set of DMSO-only cultures and incubation was continued for 6 h. During the 1st hr of culture, the final concentration of additions was: DMSO, 0.5% and DBuP or DBnP, 50 μM. After ROH addition (final concentration 0.33 μM), the PE concentration was 45 μM for the remainder of the 6 hr culture period. At the end of the 7 h culture period, cells were trypsinized, RNA was purified from pelleted cells, and RT-qPCR carried out as described previously [12]. *Alp1* primers: 5’-aacagaagttcgctatctgcc-3’ and 5’-tgcccaagagagaaacctgc-3’.

### Statistics

Data are presented as mean ± SEM on replicate cell cultures as indicated in the figure legends. The statistical significance between *Hoxa1* expression in cultures containing retinoid only and cultures containing retinoid and PEs was estimated by the Student’s t test using two-tailed distribution and two-sample unequal variance.

## Results

### Effect of PEs on the RSP during Short-Term Exposure

**Fig 2:**
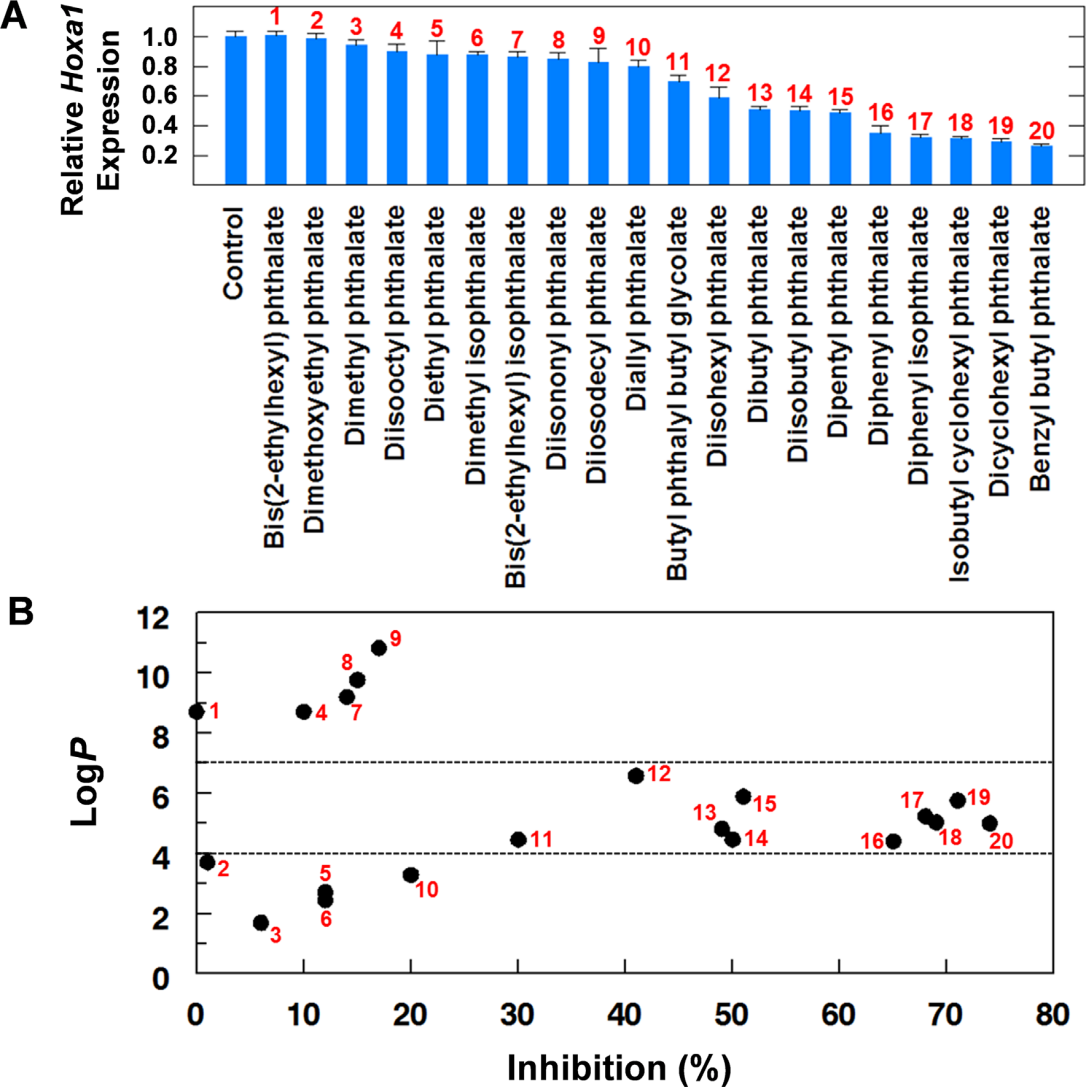
**(A) Effect of PEs on the induction of *Hoxa1* by ROH in the 7 h screen.** *Hoxa1* expression in PE-treated cultures was quantitated by RT-qPCR on cell lysates [11] and normalized to *Hoxa1* expression in ROH-only treated control cultures. The data are arranged according to increasing relative inhibitory activity. Values are Means ± SEM; n ≥ 8. **(B) Relationship between log*P* and RSP inhibition by PEs for data shown in A.** Horizontal dashed lines bracket the log*P* range containing the most inhibitory PEs. Log*P*s are calculated values obtained from the ChemSpider web site (http://www.chemspider.com).

Initially, 20 phthalate diesters representing a variety of structures were screened at a single concentration of 45 μM in the 7-h assay (Fig 1B). The compounds are arranged in Fig 2A according to their relative inhibitory activity; chemical structures are shown in S1 Fig. No evidence for cytotoxicity was detected in the MTT assay (data not shown). Of the 20 PEs, 10 inhibited the retinol signaling pathway by 30% or greater. The most potent inhibitors, #s 16–20, contained benzyl, cyclohexyl, or phenyl groups. These compounds clustered in a range of inhibitory activity between 65 and 75%. No significant difference in inhibitory activity was observed between phenyl groups in orthophthalate or isophthalate configurations (#s 16 and17, respectively). PEs containing C4-C6 alkyl ester chains (#s 12–15) were also potent pathway inhibitors, however, they were less potent than the PEs carrying ring structures.

Because different substituents can influence lipophilicity and, therefore, cellular uptake and inhibitory activity, the data were plotted to determine the relationship between log*P* and pathway inhibition (Fig 2B). Compounds with the highest inhibitory activity, >40% (#12–20), had log*P* values between 4 and 7. For compounds that inhibited the pathway by approximately 50% or greater (#13–20) the log*P* range narrowed to 4–6. PEs with very high lipid solubility (#s1, 4, 7–9) were ineffective pathway inhibitors as were the more water soluble compounds (#s 2, 3, 5, 6, 10). PEs containing aryl or cycloalkane groups (#s 16–20) had similar pathway inhibitory activity which is evident in the clustering shown in Fig 2B. This was also the case for the most inhibitory alkyl esters (#s 13–15). An interesting linear relationship between increasing log*P* and increasing pathway inhibitory activity was also observed (#s 3, 5, 6, 10, 11, 12).

### Effect of PEs on the RSP during Long-Term Exposure

**Fig 3:**
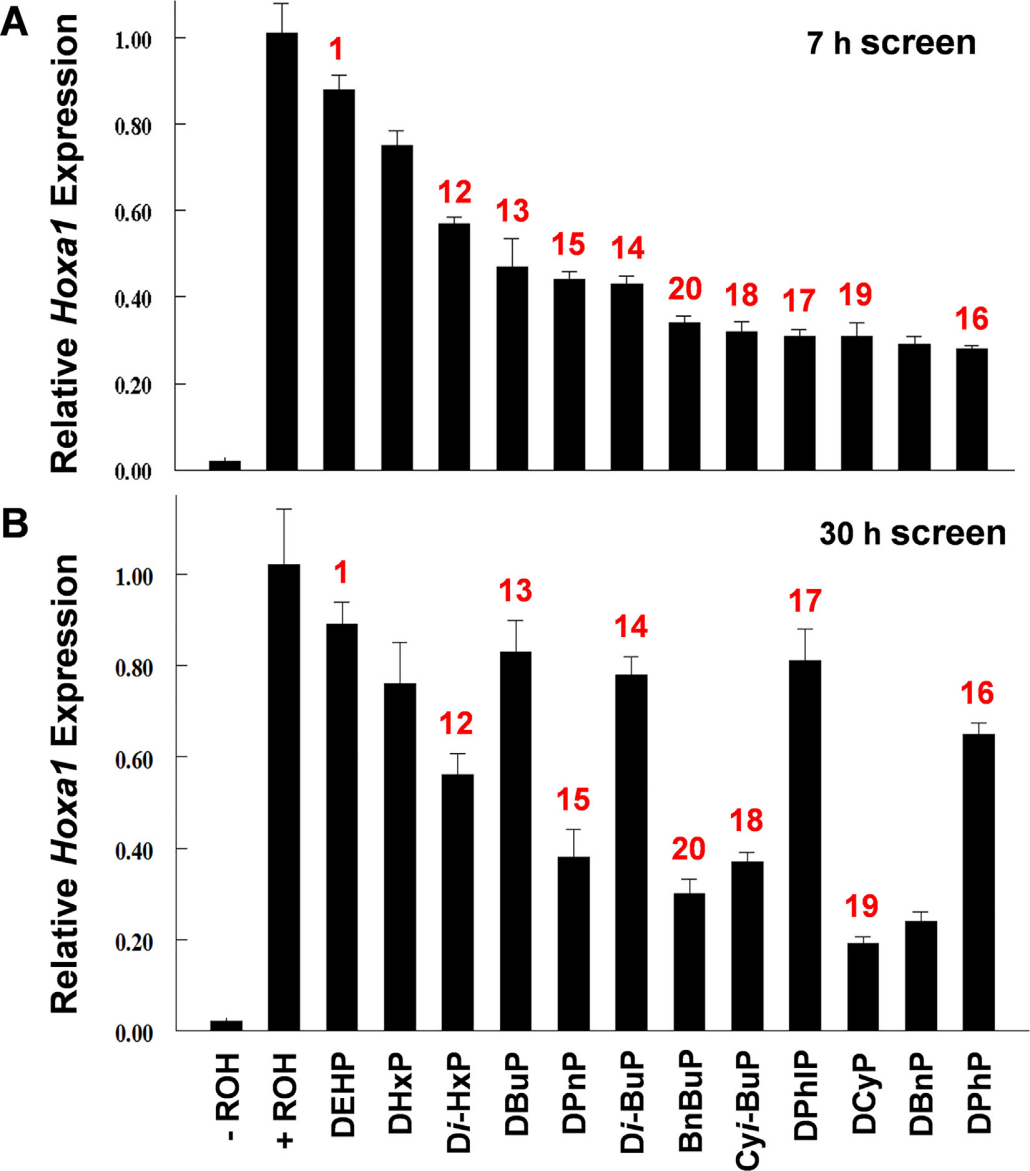
Comparison of short-term and long-term effects of PEs on the induction of *Hoxa1* expression by ROH. The same compound numbering used in Fig 2 is used in Fig 3. Compounds are arranged in Fig 3A according to their relative inhibitory activity and that format is maintained for the 30 h assay (Fig 3B). The screens were carried out on PE #s 1, 12–20 (see Fig 2A) plus DHxP and DBnP which were not screened in Fig 2 and, therefore, are not numbered. (A) 7 h screen; (B) 30 h screen; n = 4.

It was of interest to know the stability of the inhibitory response to these compounds in vitro; e.g., is inhibitory activity lost during extended time in culture which may indicate cellular metabolism or chemical instability, and what chemical structures give a more sustained response than others? To assess the effects of longer exposure in culture, cells were exposed for 30 h to the most potent inhibitory PEs (#s12-20) identified in Fig 2. Along with the 30 h exposure assay, we repeated the 7 h exposure assay for PE #s 12–20 for comparison with data in Fig 2 to assess reproducibility. Although DEHP did not significantly inhibit the pathway (Fig 2), it and dihexyl phthalate (DHxP) were added to compare with the effect of D*i*-HxP (# 12) which inhibited the pathway by ca. 40% (Fig 2). Dibenzyl phthalate (DBnP) was also added for comparison to BnBuP (# 20). The data for the 7 h and 30 h exposure assays are shown in Fig 3. The same compound numbering used in Fig 2 is used in Fig 3. Compounds are arranged in Fig 3A according to their relative inhibitory activity and that format is maintained for the 30 h assay (Fig 3B). As in the first 7 h screen (Fig 2), PEs containing aryl and cycloalkane groups were the most potent pathway inhibitors (Fig 3A). The extent of inhibition, clustering around 70%, by these compounds was the same as that seen in Fig 2. The aryl-containing diester, DBnP, clustered with this group (Fig 3A). The alkyl esters (#s12-15) also showed the similar inhibitory effect (Fig 3A) seen in Fig 2. Long-term exposure (30 h) to PEs showed the same inhibitory activity for most compounds with the notable exception of the butyl phthalates, DBuP and D*i*-BuP, and the phenyl phthalates, DPhP and DPhIP. Although these four PEs showed no evidence of cytotoxicity in the MTT assay, they showed significant loss of inhibitory activity after prolonged culture suggesting that they may be less stable in culture and/or are metabolized by the cells. With the exception of these four PEs and DCyP, which did show evidence of cytotoxicity in the MTT assay, the other compounds gave essentially the same level of pathway inhibition during the 30 h culture period as during 7h culture.

### Relative Effects of Phthalate Monoesters and Diesters on the RSP

PE-induced malformations in the male rat reproductive system are thought to be caused by the monoester metabolite of the diester compound [21]. In this in vitro test system, however, some known PE reproductive toxicants were active inhibitors of retinol signaling as diesters (Figs 2 and 3) while some were inactive. DEHP is a potent reproductive toxicant in male rats exposed in utero during late gestation but was inactive as an inhibitor of the RSP in this in vitro test system (Figs 2 and 3). It was of interest, therefore, to compare the relative inhibitory activities of some phthalate diesters with their cognate monoesters.

**Fig 4:**
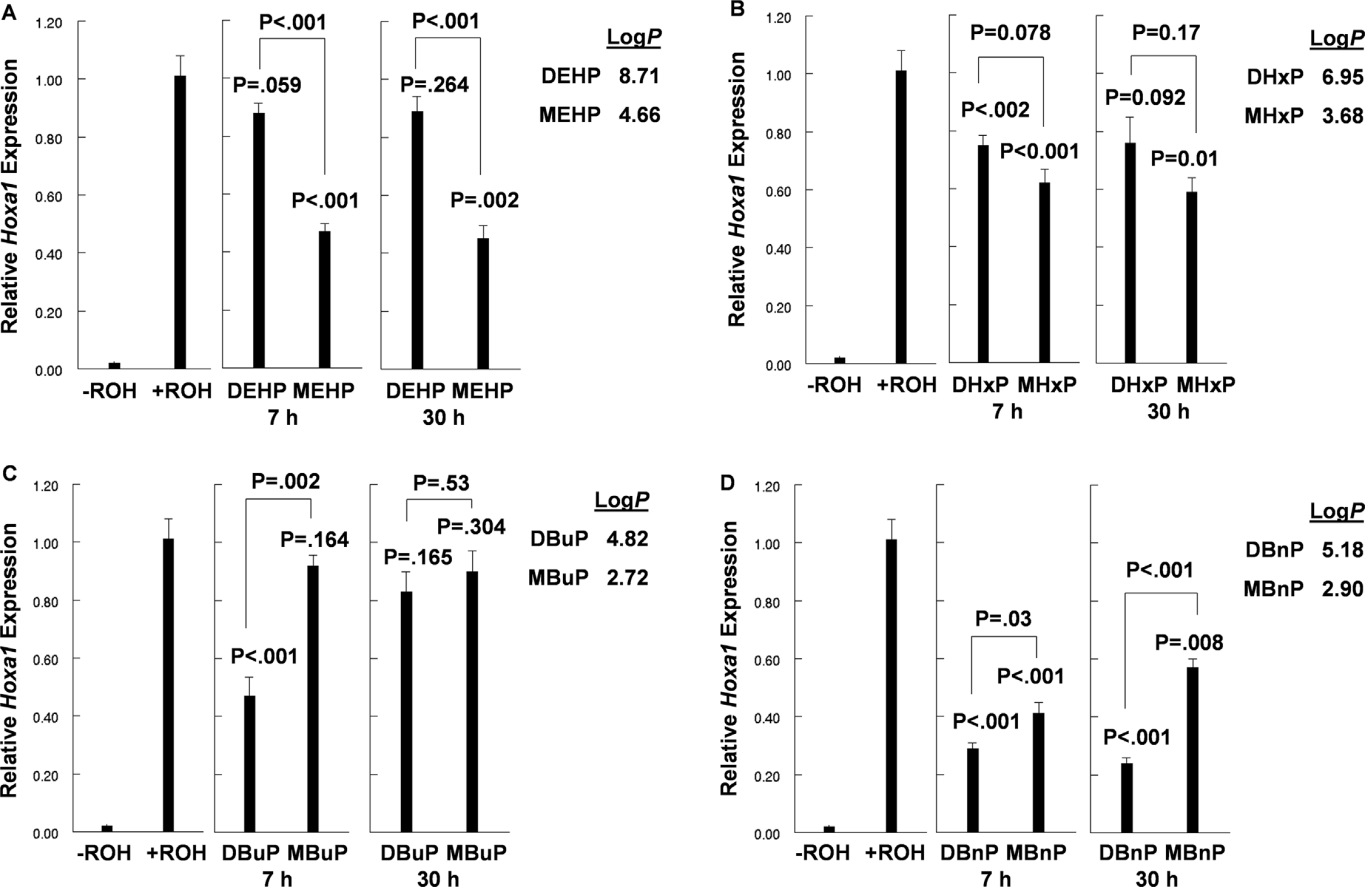
Comparison of the effects of phthalate diesters and cognate monoesters on the induction of *Hoxa1* expression by ROH. P values for phthalate effects on *Hoxa1* expression compared to +ROH controls are indicated at the top of each bar. n = 4.

A comparison of the effects of DEHP and its monoester, MEHP, is shown in Fig 4A. Although DEHP did not cause significant inhibition, MEHP significantly inhibited the pathway during both short- and long-term exposure periods. Unlike DEHP, the diester DHxP showed limited, but significant, pathway inhibition during both short-term and long-term exposure, as did its monoester, MHxP (Fig 4B). Although the monoester appeared to be more inhibitory than the diester, the difference was not significant. Again, there was no apparent difference during short or long-term culture in inhibitory activity by the diester or monoester. The levels of pathway inhibition by the C6-containing alkyl chain compounds MHxP and D*i*-HxP (cpd #12 in Fig 2A and 2B), were comparable (approximately 40%) but were less than the inhibition caused by C6-containing MEHP which carries an ethyl group on the two carbon of the alkyl chain.

A comparison of the inhibitory activities of DBuP and its monoester, MBuP, showed the monoester to be inactive as an inhibitor of the RSP (Fig 4C). Conversely, DBuP, significantly inhibited the pathway during short-term exposure but lost that ability after prolonged culture suggesting metabolism of the diester during long-term culture (Fig 4C). It is again noted that the log*P* for DBuP falls within the range occupied by other inhibitors of the RSP while the inactive monoester falls outside of this range. The loss of inhibitory activity by DBuP during long-term culture was also observed for D*i*-BuP, DPhP and DPhIP (Fig 3).

Both DBnP and MBnP significantly inhibited the pathway during short- and long-term culture (Fig 4D). The difference in inhibitory activity between the diester and monoester during short-term culture was small with the diester slightly more inhibitory. This difference increased markedly during long-term culture suggesting metabolism of the monoester to inactive metabolites. Again we note the correlation between log*P* and RSP inhibitory activity. It is worth noting that, with the exception of DHxP and MHxP (Fig 4B), the most inhibitory PE of each diester- monoester pair in Fig 4 had a log*P* value between four and six while the value of its less inhibitory partner fell considerably outside of this range.

### Phthalate-Sensitive Steps in the RSP

To elucidate the possible mechanisms through which PEs interfere with the RSP, experiments were carried out to identify where in the pathway PEs interfere. Two PE structures, representative of the two classes of PEs screened here, the alkyl diester, DBuP, and the aryl diester, DBnP, were tested as inhibitors of *Hoxa1* induction by ROH, RAL, and RA, to determine if PE-sensitive steps in the RSP could be identified. To account for the approximately 150-fold greater potency of RA as an inducer of *Hoxa1* expression compared to ROH[12] and RAL (Chen and Reese, unpublished data), the concentrations of the three retinoids were adjusted to give the same increase (approximately 30-fold) in *Hoxa1* expression during a 6 h treatment period.

**Fig 5:**
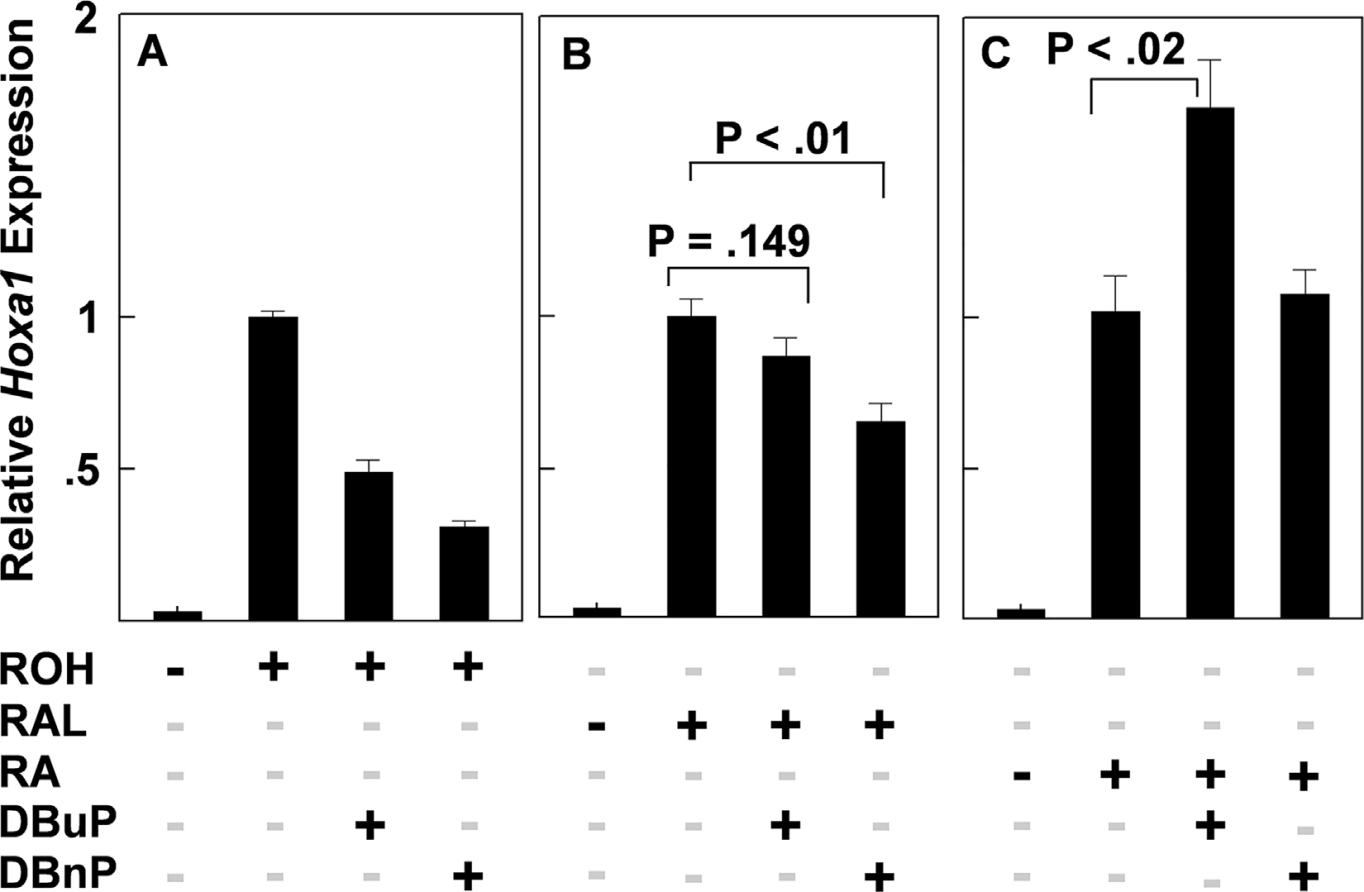
Effects of DBuP and DBnP on *Hoxa1* induction by ROH, RAL, and RA. The final ROH and RAL concentration were 0.3 μM and the RA concentration was 2.0 nM. n = 4.

Consistent with data already shown (see Figs 3, 4C and 4D), DBuP and DBnP were potent inhibitors of ROH-induced *Hoxa1* expression (Fig 5A) indicating that both compounds target essential processes associated with the first oxidative step in the retinol signaling pathway. When RAL was used to induce *Hoxa1* expression, DBuP did not significantly inhibit induction indicating that the target (or targets) of DBuP inhibitory action is associated primarily, or exclusively, with the first step in the pathway (Fig 5B). DBnP, however, significantly inhibited induction of *Hoxa1* by RAL suggesting that this aryl-containing PE may have additional targets. Finally, neither compound interfered with induction by RA indicating that neither compound interferes with post RA synthesis events that are necessary for the upregulation of *Hoxa1* expression (Fig 5C). The observation that the level of *Hoxa1* expression that was induced by RA in the presence of DBuP was greater than that induced by RA alone has been observed with some butyl-containing compounds (see Discussion).

### DBuP and DBnP Interfere with the RSP in C3H10T1/2 Mesenchymal Stem Cells

**Fig 6:**
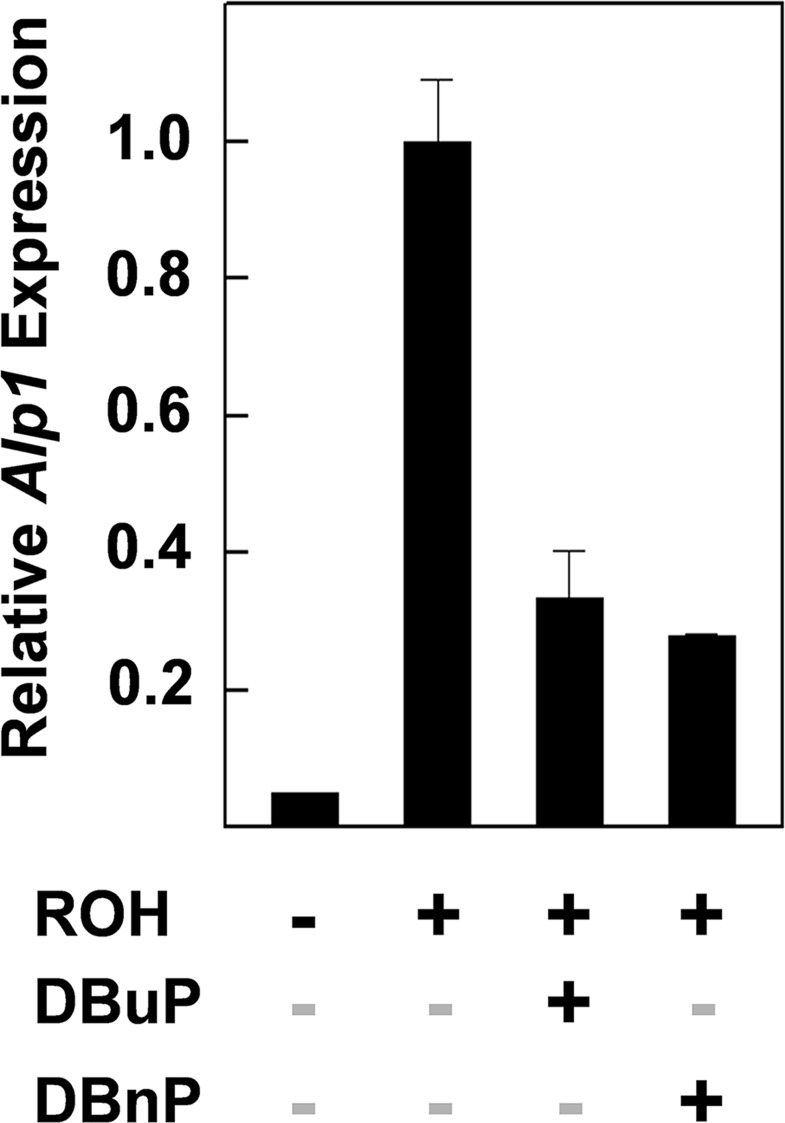
Effects of DBuP and DBnP on the induction of *Alp1* expression by ROH in C3H10T1/2 cells. 7 h screen. The final PE and ROH concentrations were 45 μM and 0.3 μM respectively. n = 2.

An additional cell type was evaluated to determine if the effects of PEs on the RSP in pluripotent P19 cells is unique to that cell type or whether other cell types give a similar response to PEs. The multipotent mesenchymal stem cell, C3H10T1/2 [24], which differs considerably from the pluripotent P19 cell in differentiation potential, can differentiate into a number of mesodermal cell types including osteoblasts, chondrocytes, adipocytes and myocytes [25]. The osteoblast marker enzyme, tissue nonspecific alkaline phosphatase (ALP1), which is essential for bone mineralization, is induced in C3H10T1/2 cells by RA [23]. It is not known, however, if this cell line has a RSP and will support the induction of *Alp1* mRNA synthesis by ROH. We tested the ability of ROH to induce *Alp1* in this cell line, which would be evidence for a RSP, and whether DBuP and/or DBnP interfere with induction, which is diagnostic of PE interference with the RSP. ROH induced an approximately 20-fold increase in *Alp1* mRNA expression during a 6 h treatment period and both PEs significantly inhibited this increase (Fig 6) indicating that the C3H10T1/2 cell, like the P19 cell, has a functional RSP which can be inhibited by PEs.

## Discussion

In vertebrate species, a tightly regulated RSP is essential for normal embryonic development and maintenance of cellular phenotype in adult tissues. Disruption of the pathway can be toxic to the fetus and adult organism. Using an in vitro screen developed for detecting chemicals that interfere with the RSP [11], we have identified multiple PE structures that significantly disrupt the pathway. Of the 26 mono- and di-esters screened in this study, 13 inhibited the pathway by 40% or more. PEs with the highest inhibitory activities shared some structural and physiochemical features. They contained either C4-C6 alkyl ester chains or esters with aryl or cycloalkane groups. With the exception of MBnP, they also had log*P* values that fell in a range between 4 and 7; for PEs that inhibited the pathway by approximately 50% or greater, the log*P* range narrowed to 4–6. It should be noted that of the PEs that fell within this range, molecules containing aryl or cycloalkane esters inhibited the pathway more than PEs with alkyl esters suggesting that, while log*P* is an important descriptor of pathway inhibitory activity in this in vitro system, structure may be a more critical factor.

The significance of the association between chemical structure, lipophilicity, and pathway inhibitory activity is unknown. PEs did not appear to interfere directly with the ability of ROH (log*P* = 6.84) to traverse the plasma membrane of P19 cells since RA with the same log*P* (6.83) induced the same level of *Hoxa1*expression in the presence or absence of DBuP or DBnP indicating that neither PE interfered with RA uptake and likely did not interfere with ROH either. Inside the cell, ROH binds tightly to the soluble cellular retinol-binding protein, RBP1/CRBP1 [26], the product of the *Rbp1* gene. Because of its high affinity for ROH, RBP1 has been proposed to facilitate cellular uptake and retention of ROH [27]. It also has been proposed to function as a chaperone for controlling enzyme access to ROH [27]. Recent data show a correlation between reduced expression of RBP1and reduced synthesis of RA in human and a mouse model of endometriosis [28] indicating the dependence of RA synthesis on cellular levels of RBP1. It is possible that inhibitory PEs share a combination of structural and physicochemical properties that facilitate access to cellular compartments, for example binding to RBP1, where they can interfere with ROH metabolism. Identification of the subcellular site(s) occupied by PEs in the P19 cell may help to elucidate the mechanism(s) of RSP inhibition by PEs and add to a better understanding of the pathway itself.

A more extensive analysis of PEs will be needed to determine if the most potent inhibitors of the pathway (#s 12–20, MEHP, and MBnP) all act on the first step or whether some, like DBnP, affect cellular processes associated with the second oxidative step. It is clear from this study, however, that both DBuP and DBnP interfered with the synthesis of RA (Fig 5A and 5B) and that neither compound interfered with post RA-synthesis events necessary for gene transcription (Fig 5C). It is possible that PEs may be identified in the future that interfere with post RA synthesis events and, therefore, would be detected by probing cell cultures with RA. However, it is worth noting, in light of the overwhelming number of in vitro studies that use RA as a probe relative to studies that use ROH, that an adverse effect of a chemical caused by inhibition of RA synthesis is not likely to be detected using RA as a probe and may only be detectable using ROH.

The enhanced expression of *Hoxa1* above control values that was induced by DBuP but not by DBnP (see Fig 5C) has been seen with other butyrate-containing compounds that have been tested in this screen (Chen and Reese unpublished). While this phenomenon may have other plausible explanations, we postulate that the C4 compound, butyrate, a known inhibitor of class I histone deacetylases (HDAC) [29], is particularly relevant to the DBuP effect observed here. Large co-repressor complexes bind to unliganded nuclear receptors, e.g., the retinoic acid receptors (RAR-RXR) in the absence of RA, and recruit class I histone deacetylases (HDAC) to the complex. HDACs remove acetyl groups from histone tails in the chromatin complex causing chromatin compaction and transcriptional repression [30, 31]. It is possible, therefore, that the two C4 chains of DBuP may inhibit HDAC enzyme activity associated with the co-repressor complex. This would cause transcriptional de-repression thus enhancing the level of *Hoxa1* expression above that initiated by the concentration of RA used in this study (Fig 5C) which was less than the RA concentration required for maximal expression[12]. We have observed that butyrate, and another HDAC1 inhibitor, tricostatin A, significantly upregulate the expression of *Hox* genes, including *Hoxa1*, in P19 cells (Reese, unpublished data). It should also be pointed out that if DBuP-induced overexpression of *Hoxa1* observed in this study is caused by butyrate, then other members of the nuclear receptor family may be similarly affected by C4-containing PEs.

Although DEHP did not significantly inhibit the pathway, its monoester, MEHP was inhibitory suggesting that P19 cells lack the ability to metabolize DEHP to MEHP, even during long-term culture and that the monoester, or a metabolite, is an inhibitor of the retinol signaling pathway. It is interesting that MEHP inhibitory activity was the same for both short-term and long-term culture period suggesting that MEHP or an active metabolite are stable in culture for extended periods of time. It is notable that the log*P* value (4.66) for MEHP falls within the range occupied by the most potent PE inhibitors of the RSP (Fig 2B) while the value for the inactive DEHP falls considerably outside of this range. This was not the case with other diesters (#s 12–20 and DBnP) which significantly inhibited the pathway. Whether these diesters interfered directly with the pathway or first required metabolism to the monoester, or a metabolite, will need to be answered by metabolite analysis of PE-exposed P19 cells. During short-term culture, DBuP appeared to act directly to interfere with the pathway since its monoester metabolite, MBuP, was not inhibitory. Furthermore, after long-term culture DBuP lost inhibitory activity suggesting that it was metabolized to the non-inhibitory monoester or another metabolite. The lack of pathway inhibitory activity by MBuP in this in vitro system is contrary to results from in vivo studies which conclude that the monoester is the active form responsible for the effects of DBuP [32]. This lack of inhibition by MBuP may be related to factors unique to the in vitro system and further emphasizes the importance of metabolite analysis in understanding how phthalates disrupt the RSP in vitro which also may shed light on in vivo mechanisms. Since DPhP and DPhIP also lost much of their inhibitory activity during long-term culture, it would be interesting to know if their monoesters, like MBuP, lack inhibitory activity and if in utero exposure to DPhP and DPhIP cause reproductive system malformations in the male rat fetus.

RA was shown previously to be a potent inducer of ALP1 (tissue non-specific alkaline phosphatase) in mouse C3H10T1/2 cells [23]. It was not known, however, if ROH could induce *Alp1* mRNA synthesis in this cell line which would be evidence for metabolism of ROH to RA and the presence of a functional RSP in this multipotent mesenchymal stem cell. Both DBuP and DBnP significantly inhibited the ROH-induced synthesis of *Alp1* in C3H10T1/2 cells in this study. The inhibition of the RSP by PEs, therefore, is not unique to the P19 cell and probably occurs in other cell types that have a functioning pathway in the embryo and in fully differentiated cells in adult organisms.

The inhibition of RA synthesis caused by PEs in this in vitro study is the functional equivalent of VAD. Vitamin A deficiency can be caused by depriving an organism of vitamin A needed for RA synthesis or by inhibiting the synthesis of RA from vitamin A. In both cases, RA deficiency is the basis of malformations in the developing embryo [13, 14]. It has been known for almost a century that VAD causes a spectrum of reproductive system malformations, including dysgenesis of the seminiferous tubules, seminal vesicles, epididymis, prostate, genital tubercle (hypospadias), and cryptorchidism, in the reproductive system of male rat fetuses [33–35]. Next to eye defects, malformations of the genital ducts were the most frequently observed adverse effects of VAD[36]. One study [35] described the effects of VAD as causing complete testicular atrophy in the absence of any impairment of testicular circulation or supporting tissues indicating that the adverse effect on testis development was directly on the testis and not an indirect effect.

The types of adverse effects caused by VAD and PEs in the development of male rat reproductive system are strikingly similar. In addition, PEs found to be the most potent inhibitors of the RSP in this in vitro study (DPnP, DCyP, DBuP, BnBuP, D*i*-BuP) are also some of the most effective inducers of malformations in the male rat reproductive system [37–41]. This correlation between PEs that interfere with the pathway in vitro and PEs that cause malformations in utero may involve more than the five PEs listed here since four additional potent in vitro inhibitors of the pathway, DBnP, DPhP, DPhIP, and Cy*i*-BuP, have not, to our knowledge, been tested in vivo. Moreover, Cy*i*-BuP is closely related structurally to DCyP which inhibits the pathway in vitro and causes malformations in utero [38]. In light of the malformations caused by VAD and PEs in the male rat, it is also worth noting that the ovaries of female rats from VAD mothers did not appear to be adversely affected [35, 36] nor apparently were the ovaries of females exposed in utero to Pes [9]. The significance of this difference between the response of male and female rats to both VAD and PEs is unknown.

In addition to the adverse effects of VAD on the development of the male reproductive system described in previous studies, recent mechanistic studies provide additional evidence for the important role RA plays in the development and maintenance of the male reproductive system. RA has been shown to play a role in Sertoli cell differentiation and function [42, 43] and to regulate the expression of *Stra8* (*stimulated by retinoic acid gene 8*), the gene that controls entry into meiosis and, therefore, is essential for sperm maturation [44]. Recent studies also show that RA is necessary for the development of the male genital tubercle, the anlage of the penis [45]. Furthermore, it may play a role in testicular decent by regulating the expression of Rxfp2/Lgr8 [46], the receptor for the Leydig cell hormone, INSL3, thought to be necessary for testicular decent [6]. In addition, *Rdh10* and *Aldh1a2/Raldh2*, the genes that code for the enzymes that catalyze the first and second oxidative steps in RA synthesis, respectively, show enhanced stage-specific patterns of expression in the genital tubercle, Wolffian duct, and Wolffian duct-derived tissues during mouse embryogenesis [45, 47–49] further highlighting the importance of RA synthesis in the development of these male reproductive structures.

**Fig 7:**
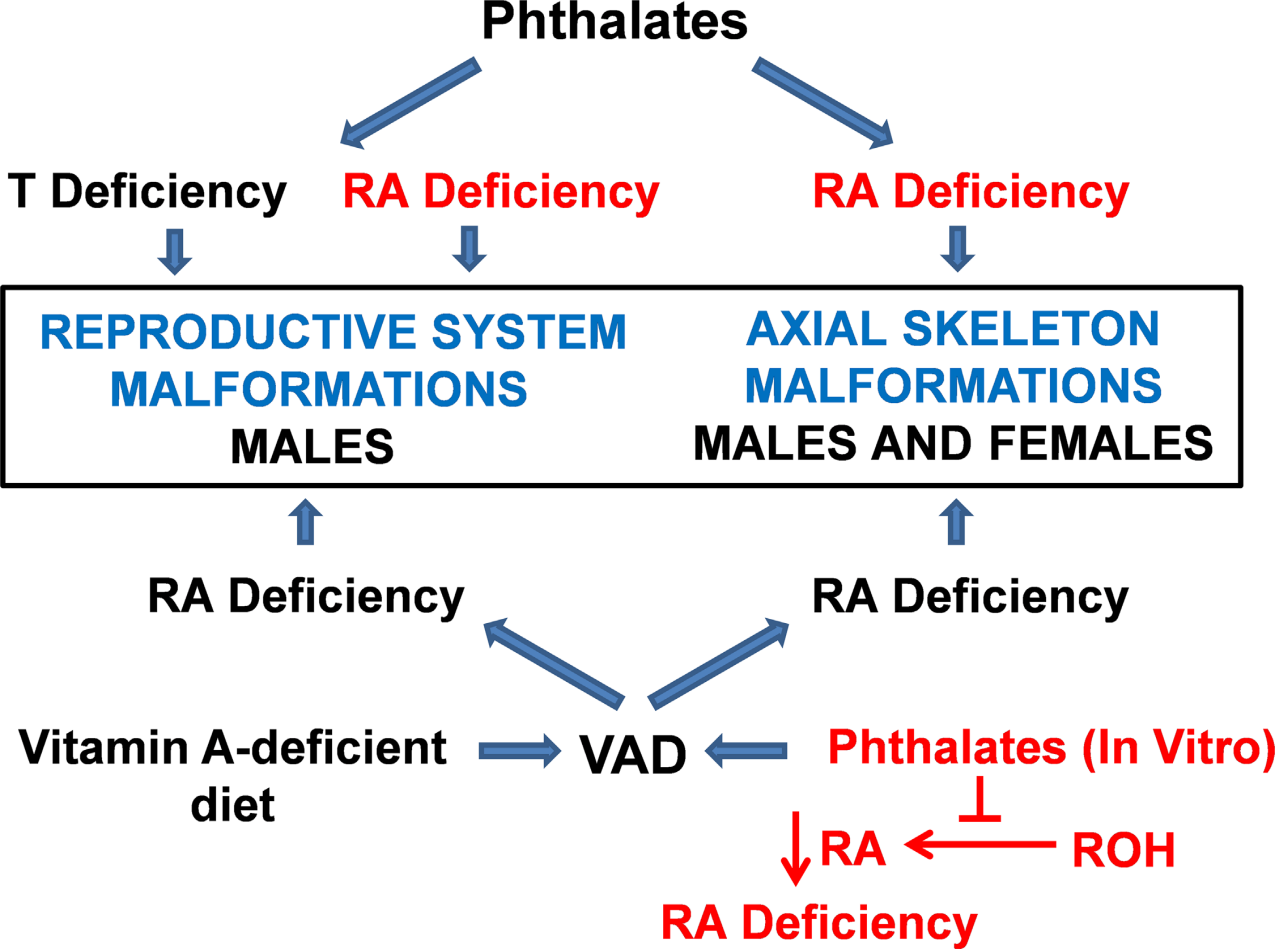
Synopsis of connections between fetal malformations induced by PEs and RA deficiency. Gestational exposure to some phthalates or to a VAD (vitamin A deficient/RA deficient) diet cause a characteristic spectrum of malformations (dysgenesis of seminiferous tubules, seminal vesicles, epididymis, prostate, genital tubercle/hypospadias, and cryptorchidism) in the reproductive system of the male rat fetus; phthalates or VAD also cause malformations in the axial skeleton of both sexes. PE-mediated fetal testosterone (T) deficiency is generally considered to be the cause of male reproductive system malformations. Given that PEs also caused cellular RA deficiency in this in vitro study, PE-mediated RA deficiency is hypothesized (indicated in red) to be an additional cause of malformations in the male rat reproductive system and axial skeleton in utero.

We propose, therefore, that the reproductive system malformations seen in male rat fetuses exposed in utero to PEs are due, in part, to disruption of the RSP and resultant inhibition of RA synthesis. This is based on (1) the similarities in the types of malformations induced in the male reproductive system in utero by both PEs and VAD; (2) data presented here showing that PEs cause the equivalent of VAD in vitro by interfering with the synthesis of RA; (3) PEs that are the most potent inhibitors of RA synthesis in vitro are also the most effective inducers of reproductive system malformations in utero; and (4) mechanistic studies documenting the essential role that RA synthesis plays in male reproductive system development and function. This hypothesis is further supported by studies showing that in utero exposure to DBuP during critical periods in development led to malformations, suggestive of homeotic transformations, in the axial skeleton of male and female rats [32] and that a VAD diet also causes a pattern of homeotic transformations in the rat axial skeleton [33, 50]. It should also be noted that excessive levels of RA also induce distinct patterns of homeotic transformations in the mouse axial skeleton [17, 51]. The fact that PEs cause malformations in two organ systems, reproductive and skeletal, that are dependent on RA for normal development suggests that PEs have the potential to induce the equivalent of VAD in other embryonic or adult cells that require RA synthesis for maintenance of phenotype. Fig 7 is a summary depicting the relationships between malformations caused by phthalates and VAD discussed above.

We are not proposing that PE-induced VAD is an alternative mechanism for the cause of male reproductive system malformations. Nor do we suggest that lowered levels of T are not a cause of malformations in male reproductive tissues. We think, however, that PE inhibition of RA synthesis during early stages of male reproductive system development may contribute to the severity or extent of malformations caused by low T levels. RA can play permissive and instructive roles in development. It is thought to create permissive conditions for forelimb induction [52] and given the mechanistic similarity in strategies used in the development of limbs and external genitalia [53], RA also may play a similar role in the development of some male reproductive structures. During development of the mouse genital tubercle (GT), RA signaling plays a role in regulating the Sonic hedgehog signaling pathway [45] which is essential for both initial and sexually dimorphic development of the male external genitalia [54]. Hedgehog signaling has been suggested to promote masculinization of the external genitalia by facilitating androgen responsiveness in the GT mesenchyme and it should be noted that androgen alone does not appear to be sufficient for GT development in the absence of RA-regulated Shh signaling [54].

If PE-induced malformations in the male rat reproductive system are caused, in part, by the inhibition of RA synthesis, it may be possible to lessen these effects by administering RA to PE-treated pregnant animals. RA can support the growth of rats raised on a vitamin A deficient diet [33] and RA has been shown to rescue the adverse effects of experimentally induced VAD [55, 56]. Evidence for amelioration of the adverse effects of PEs by RA supplementation in the rat model may have therapeutic relevance to studies in humans that show an association between PE exposure and adverse health effects in cells and tissues that are dependent on retinol signaling for maintenance of normal phenotype.

## Supporting Information

S1 Fig Chemical structures of phthalate diesters used in this study. (TIF)

S1 Table CAS number, commercial source, and chemical purity of all phthalates used in this study. (DOCX)

## Abbreviations

ALDH1A2: aldehyde dehydrogenase 1A2/retinaldehyde dehydrogenase2/raldh2
BnBuP: benzyl butyl phthalate
Cy*i*-BuP: cyclohexyl isobutyl phthalate
DBnP: dibenzyl phthalate
DBuP: dibutyl phthalate
DCyP: dicyclohexyl phthalate
DEHP: bis(2-ethylhexyl) phthalate
DHRS3: dehydrogenase/reductase (SDR family) member 3
DHxP: dihexyl phthalate
D*i*-BuP: diisobutyl phthalate
D*i*-HxP: diisohexyl phthalate
DPhP: diphenyl phthalate
DPhIP: diphenyl isophthalate
DPnP: dipentyl phthalate
GT: genital tubercle
MEHP: mono(2-ethylhexyl) phthalate
MHxP: monohexyl phthalate
MBuP: monobutyl phthalate
MBnP: monobenzyl phthalate
PE: phthalate ester
RA: all-*trans*-retinoic acid
RAL: retinaldehyde
RXR/RAR: retinoic acid receptor heterodimers
RDH10: retinol dehydrogenase 10
RSP: retinol signaling pathway
ROH: retinol (vitamin A)
SAR: structure-activity relationship
T: testosterone
VAD: vitamin A deficiency
