# Supplementary figures and images for: Disruption of Retinol (Vitamin A) Signaling by Phthalate Esters: SAR and Mechanism Studies
Source: PLoS One. 2016 Aug 17;11(8):e0161167. doi: 10.1371/journal.pone.0161167 (PMC4988654; doi:10.1371/journal.pone.0161167)

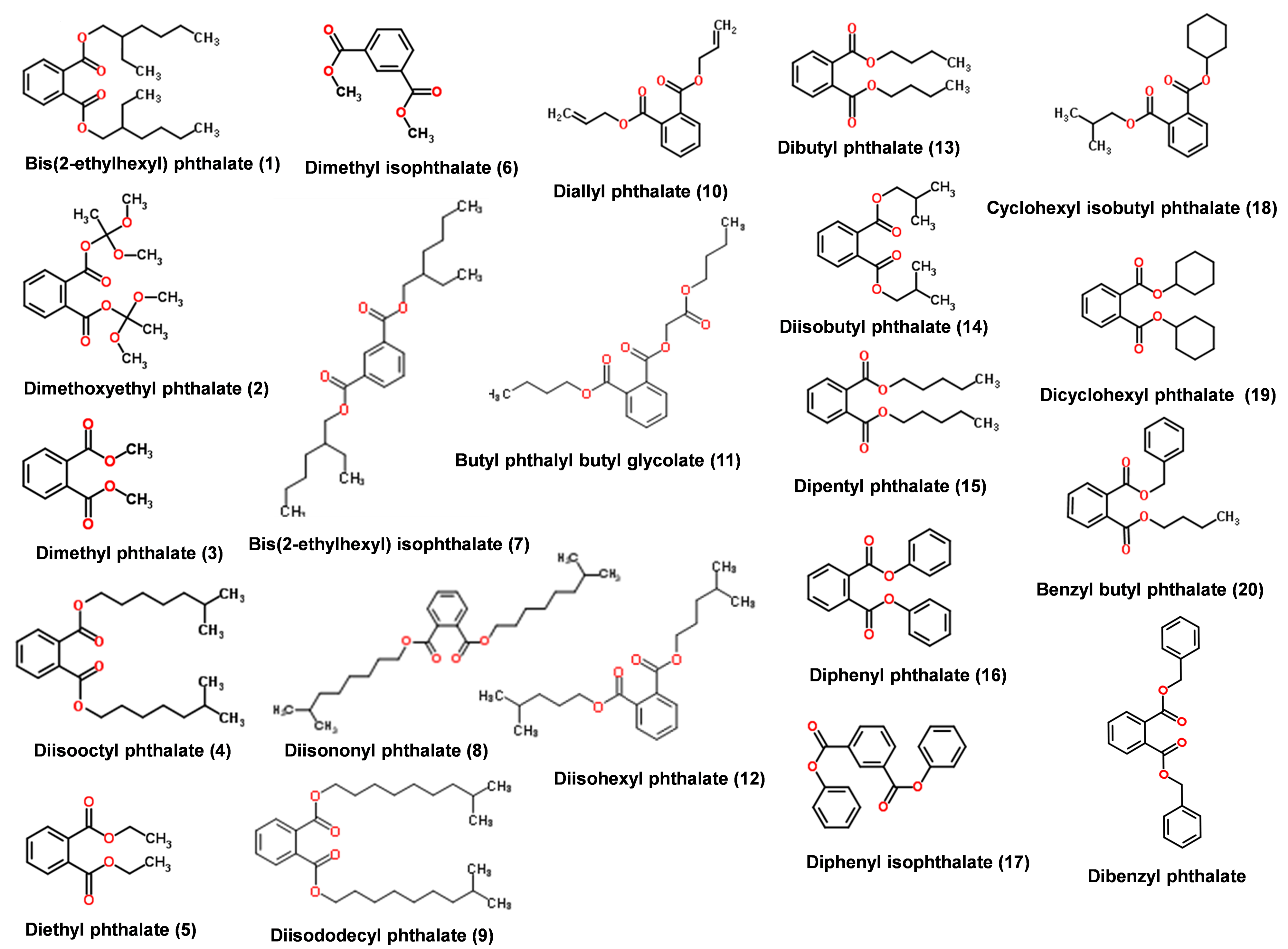

Supplement: S1 Fig — (TIF) [file pone.0161167.s001.tif]
