# Supplementary material for: Disruption of Retinol (Vitamin A) Signaling by Phthalate Esters: SAR and Mechanism Studies
Source: PLoS One. 2016 Aug 17;11(8):e0161167. doi: 10.1371/journal.pone.0161167 (PMC4988654; doi:10.1371/journal.pone.0161167)
Supplement: S1 Table — (DOCX) [file pone.0161167.s002.docx]

**S1 Table. CAS number, commercial source, and chemical purity of all phthalates used in this study**

| **Chemical Name** | **CAS #** | **Source** | **Purity (%)** |
| --- | --- | --- | --- |
| Bis(2-ethylhexyl) phthalate | 117-81-7 | SA | 99 |
| Dimethoxyethyl phthalate | 117-82-8 | US | 99 |
| Dimethyl phthalate | 131-11-3 | US | 99 |
| Diisooctyl phthalate | 27554-26-3 | US | IM/T |
| Diethyl phthalate | 84-66-2 | US | 99 |
| Dimethyl isophthalate | 1459-93-4 | US | 95 |
| Bis(2-ethylhexyl) isophthalate | 137-89-3 | US | 99 |
| Diiosononyl phthalate | 68515-48-0 | US | IM/T |
| Diisodecyl phthalate | 26761-40-0 | US | IM/T |
| Diallyl phthalate | 131-17-9 | US | 99 |
| Butyl phthalyl butyl glycolate | 85-70-1 | US | 92 |
| Diisohexyl phthalate | 6851-5-4 | US | T |
| Dibutyl phthalate | 84-74-2 | US, SA | 94, 99 |
| Diisobutyl phthalate | 84-69-5 | US | 98 |
| Dipentyl phthalate | 131-18-0 | SA | 99 |
| Diphenyl phthalate | 84-62-8 | US | 99 |
| Diphenyl isophthalate | 744-45-6 | US | 99 |
| Cyclohexyl isobutyl phthalate | 5334-09-8 | US | T |
| Dicyclohexyl phthalate | 84-61-7 | US | 97 |
| Benzyl butyl phthalate | 85-68-7 | SA | 98 |
| Dibenzyl phthalate | 523-31-9 | SC | 97 |
| Dihexyl phthalate | 85-78-3 | TCIA | 98 |
| Mono(2-ethlhexyl) phthalate | 25425-73-4 | SA | 95 |
| Monobutyl phthalate | 5423-38-1 | SA | 97 |
| Monobenzyl phthalate | 2528-16-7 | SA | 98 |
| Monohexyl phthalate | 24539-57-9 | TCIA | 98 |
| **Symbols:** SA, Sigma-Aldrich; SC, Santa Cruz Biotechnology; US, Ultra Scientific; TCIA, TCI America; IM, Isomeric Mixture; T, Technical Grade | | | |
